# Supplementary material for: At Least Three Doses of Leading Vaccines Essential for Neutralisation of SARS-CoV-2 Omicron Variant
Source: Front Immunol. 2022 May 17;13:883612. doi: 10.3389/fimmu.2022.883612 (PMC9152325; doi:10.3389/fimmu.2022.883612)
Supplement: Supplementary Table 1 — Comparison for NT50 values on 2nd-2wk [file Table_1.docx]

**Supplementary Table S1: Comparison for NT50 values on 2^nd^-2wk**

| Comparison | Variant | Estimate  (‘adjusted’ p-value) |
| --- | --- | --- |
| Pfizer vs Moderna | VIC31 | p > 0.100 |
| Pfizer vs Moderna | Omicron | p > 0.100 |
| Pfizer vs Moderna | Delta | p > 0.100 |
