# Supplementary material for: At Least Three Doses of Leading Vaccines Essential for Neutralisation of SARS-CoV-2 Omicron Variant
Source: Front Immunol. 2022 May 17;13:883612. doi: 10.3389/fimmu.2022.883612 (PMC9152325; doi:10.3389/fimmu.2022.883612)
Supplement: Supplementary Table 2 — One-way ANOVA results for vaccines, variants, sex, age and day post vaccination/boosters. [file Table_2.docx]

**Supplementary Table S2: One-way ANOVA results for vaccines, variants, sex, age and day post vaccination/boosters**

| **Vaccine** | **Variable** | **F-value** | **p-value** | **Tukey’s HSD post hoc Comparison**  **(‘adjusted’ p-value)** |
| --- | --- | --- | --- | --- |
| Pfizer | Sex | 0.002 | >0.100 | No significant difference |
|  | Age | 0.667 | >0.100 | No significant difference |
|  | Day post vaccination/booster | 119.9 | <0.0001 | Pre 1^st^ Dose – 2wk Post-2^nd^ Dose p < 0.0001 |
|  |  |  |  | Pre 1^st^ Dose – 6mo Post-2^nd^ Dose p < 0.01 |
|  |  |  |  | Pre 1^st^ Dose – 2wk Post-3^rd^ Dose p < 0.0001 |
|  |  |  |  | 2wk Post 2^nd^ Dose – 6mo Post-2^nd^ Dose p < 0.0001 |
|  |  |  |  | 2wk Post 2^nd^ Dose – 2wk Post-3^rd^ Dose p < 0.0001 |
|  |  |  |  | 6mo Post 2^nd^ Dose – 2wk Post-3^rd^ Dose p < 0.0001 |
|  | Variant | 15.86 | <0.0001 | Delta – Omicron p < 0.0001 |
|  |  |  |  | VIC31 – Omicron p < 0.0001 |
| Moderna | Sex | 0.104 | >0.100 | No significant difference |
|  | Age | 0.074 | >0.100 | No significant difference |
|  | Day post vaccination/booster | 64.56 | <0.0001 | Pre 1^st^ Dose – 2wk Post-2^nd^ Dose p < 0.0001 |
|  | Variant | 8.839 | <0.001 | Delta – Omicron p < 0.05 |
|  |  |  |  | VIC31 – Omicron p < 0.001 |
| AstraZeneca | Sex | 1.497 | >0.100 | No significant difference |
|  | Age | 1.401 | >0.100 | No significant difference |
|  | Day post vaccination/booster | 7.722 | <0.01 | Pre 1^st^ Dose – 2wk Post-2^nd^ Dose p < 0.01 |
|  | Variant | 5.744 | <0.01 | VIC31 – Delta p < 0.05 |
|  |  |  |  | VIC31 – Omicron p < 0.05 |
