# Supplementary material for: At Least Three Doses of Leading Vaccines Essential for Neutralisation of SARS-CoV-2 Omicron Variant
Source: Front Immunol. 2022 May 17;13:883612. doi: 10.3389/fimmu.2022.883612 (PMC9152325; doi:10.3389/fimmu.2022.883612)
Supplement: Supplementary Table 3 — Two-way ANOVA results of interactions for vaccines, variants, sex, age and day post vaccination/boosters †In two-way ANOVA the main effect for variant was statistically significant (p < 0.0001). However, the interaction effects of variant with age or sex were not statistically significant (p > 0.05) but there were statistically significant interactions between the effects of variant and day post vaccination/booster (p < 0.001). [file Table_3.docx]

**Supplementary Table S3: Two-way ANOVA results of interactions for vaccines, variants, sex, age and day post vaccination/boosters**

| **Vaccine** | **Factor 1** | **Factor 2** | **F-Value** | **p-Value** | **Tukey’s HSD post hoc Comparison**  **(‘adjusted’ p-value)** |
| --- | --- | --- | --- | --- | --- |
| Pfizer | Variant | Sex | 0.053 | >0.100 | No significant difference |
|  |  | Age | 0.018 | >0.100 | No significant difference |
|  |  | Day post vaccination/booster | 24.54 | <0.0001^†^ | Pre 1^st^ Dose – 2wk Post-2^nd^ Dose p < 0.0001 |
|  |  |  |  |  | Pre 1^st^ Dose – 6mo Post-2^nd^ Dose p < 0.0001 |
|  |  |  |  |  | Pre 1^st^ Dose – 2wk Post-3^rd^ Dose p < 0.0001 |
|  |  |  |  |  | 2wk Post 2^nd^ Dose – 6mo Post-2^nd^ Dose p < 0.0001 |
|  |  |  |  |  | 2wk Post 2^nd^ Dose – 2wk Post-3^rd^ Dose p < 0.0001 |
|  |  |  |  |  | 6mo Post 2^nd^ Dose – 2wk Post-3^rd^ Dose p < 0.0001 |
|  |  |  |  |  | Delta – Omicron p < 0.0001 |
|  |  |  |  |  | VIC31 – Omicron p < 0.0001 |
| Moderna | Variant | Sex | 0.158 | >0.100 | No significant difference |
|  |  | Age | 0.040 | >0.100 | No significant difference |
|  |  | Day post vaccination/booster | 59.91 | <0.0001^†^ | Pre 1^st^ Dose – 2wk Post-2^nd^ Dose p < 0.0001 |
|  |  |  |  |  | Delta – Omicron p < 0.05 |
|  |  |  |  |  | VIC31 – Omicron p < 0.001 |
| AstraZeneca | Variant | Sex | 0.872 | >0.100 | No significant difference |
|  |  | Age | 0.507 | >0.100 | No significant difference |
|  |  | Day post vaccination/booster | 13.00 | <0.0001^†^ | Pre 1^st^ Dose – 2wk Post-2^nd^ Dose p < 0.01 |
|  |  |  |  |  | Delta – Omicron p < 0.05 |
|  |  |  |  |  | VIC31 – Omicron p < 0.05 |

^†^In two-way ANOVA the main effect for variant was statistically significant (p < 0.0001). However, the interaction effects of variant with age or sex were not statistically significant (p > 0.05) but there were statistically significant interactions between the effects of variant and day post vaccination/booster (p < 0.001).
