# Supplementary figures and images for: At Least Three Doses of Leading Vaccines Essential for Neutralisation of SARS-CoV-2 Omicron Variant
Source: Front Immunol. 2022 May 17;13:883612. doi: 10.3389/fimmu.2022.883612 (PMC9152325; doi:10.3389/fimmu.2022.883612)

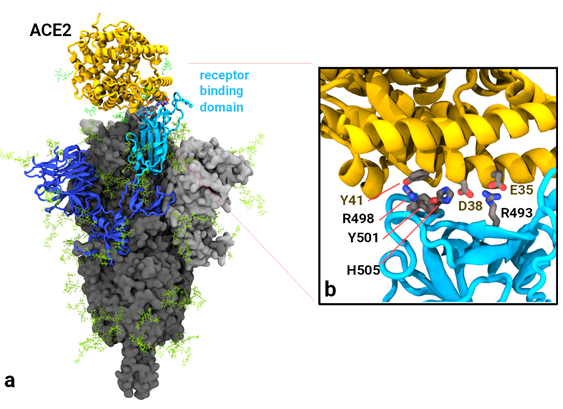

Supplement: Supplementary Figure 1 — (A) Omicron spike protein binding to ACE2 domain (shown in yellow). S1 domain shown in blues, with the receptor binding domain shown in cyan. Glycosylation is shown as green sticks. (B) close up of the ACE2 binding interface of Omicron spike RBD showing the interaction of contacting residues. [file Image_1.tif]

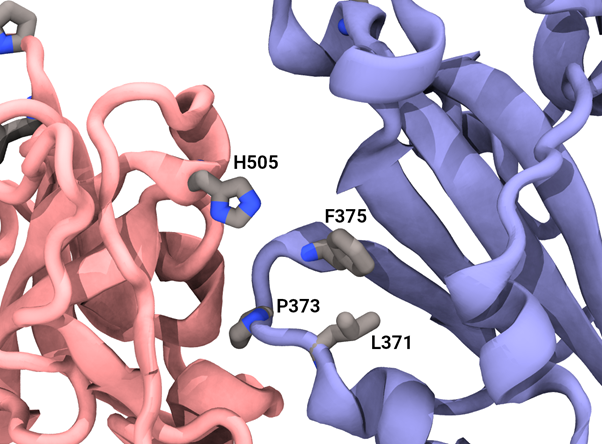

Supplement: Supplementary Figure 2 — Close up of Omicron mutations in adjacent receptor binding domains (pink and blue) in ‘down’ position showing the close proximity of residues L371, P373 and F375 on the blue domain to the adjacent H505. This complementary arrangement may have influence on the RBD ‘up’/’down’ transitions by pH dependant ionization of H505. [file Image_2.tif]
