# Supplementary material for: At Least Three Doses of Leading Vaccines Essential for Neutralisation of SARS-CoV-2 Omicron Variant
Source: Front Immunol. 2022 May 17;13:883612. doi: 10.3389/fimmu.2022.883612 (PMC9152325; doi:10.3389/fimmu.2022.883612)
Supplement: Supplementary file 6 [file DataSheet_1.docx]

**Supplementary Methods**

| **Statistical analysis using linear mixed models**  Linear Mixed Models (*lm* and *lmer*) analyses were used to investigate the interactions of age, sex, vaccine, and day post vaccination/booster with neutralising antibody titres to the three different virus variants using‘*lme4’* library in R [**1**]. To avoid, over fitting of data, we are not showing the results of *lm* and *lme* analyses. One-way and two-way ANOVA analyses showed that, for all vaccines (Pfizer, Moderna and AstraZeneca), day post vaccination/booster and virus variant were the main contributors to differences in neutralising antibody responses, while age and sex did not have significant effects.  Linear Mixed Model analysis of the variance between variables (results not shown) confirmed the ANOVA analyses which showed that day post vaccination/booster and virus variant are the major contributors to titre differences, but further shows that there was a significant difference in neutralising titres elicited by the three different vaccines, mostly due to poor responses to the AstraZeneca vaccine. This analysis model further suggests that younger individuals (aged less than 35 years) are likely to have a stronger neutralising antibody response than older individuals, but we concede that this finding might be confounded by the distribution of the ages within the vaccine groups. This might also explain the poorer responses in the AstraZeneca group because of the vaccine advice in Australia (AstraZeneca mostly used in the older age group). |
| --- |
| **Modelling using *in silico* methods**  Molecular simulations were performed using NAMD2.14 with CHARM36m forcefield [**2**] employing a TIP3 water model. The Spike model was based on the pdb structure 6VSB3 [**3**], built with additions segments predicted with AlphaFold. The Spike protein only included residues 13 to 1160 (omitting transmembrane domain, to reduce the simulation size). Glycosylation of the Spike protein was manually constructed using Visual Molecular Dynamics (VMD) guided by glycan analysis [**4**]. Omicron models were constructed in two conformations, all RBD ‘down’, and 2 RBD ‘down’ one RBD ‘up’. Both models included an ACE2 domain in the expected binding position.  Simulations were run with Periodic Boundary Conditions ‘PBCs’ using the NPT ensemble at 310K and 1 bar pressure employing Langevin dynamics. The PBCs were constant in the XY dimensions. Long-range Coulomb forces were computed with the Particle Mesh Ewald method with a grid spacing of 1 Å. 2 fs timesteps were used with non-bonded interactions calculated every 2 fs and full electrostatics every 4 fs while hydrogens were constrained with the ‘SHAKE’ algorithm. The cut-off distance was 12 Å with a switching distance of 10 Å and a pair-list distance of 14 Å. Pressure was controlled to 1 atmosphere using the NoséHoover Langevin piston method employing a piston period of 100 fs and a piston decay of 50 fs. Trajectory frames were captured every 100 ps. Simulations were performed for at least 200 nanoseconds. Trajectories were visualized and analysed using VMD [**5**]. |
| (1) R-Core-Team. *R: A Language and Environment for Statistical Computing*; R Foundation for Statistical Computing: Vienna, Austria, 2019. Available online: <https://www.r-project.org/>  (2) Huang, J. et al. CHARMM36m: an improved force field for folded and intrinsically disordered proteins. *Nature Methods* (2017) 14, 71-73, doi:10.1038/nmeth.4067  (3) Wrapp, D. et al. Cryo-EM structure of the 2019-nCoV spike in the prefusion conformation. *Science* (2020) 367, 1260-1263 doi: 10.1126/science.abb2507  (4) Watanabe Y, Allen, JD, Wrapp, J, McLellan, JS, Crispin, M. Site-specific glycan analysis of the SARS-CoV-2 spike *Science* (2020) 369(6501):330-333. doi: 10.1126/science.abb9983.  (5) Humphrey, W., Dalke, A. and Schulten, K., "VMD - Visual Molecular Dynamics", *J. Molec. Graphics*, 1996, vol. 14, pp. 33-38.41 |
